# Soft, Wirelessly Powered Humidity Sensor Based on SnO_2_ Nanowires for Wireless/Wearable Sensor Application

**DOI:** 10.3390/ma13092176

**Published:** 2020-05-08

**Authors:** Gunchul Shin

**Affiliations:** School of Materials Science and Engineering, University of Ulsan, 12 Technosaneop-ro 55 beon-gil, Nam-gu, Ulsan 44776, Korea; gunchul@ulsan.ac.kr; Tel.: +82-52-712-8067

**Keywords:** humidity, sensor, SnO_2_ nanowires, wearable, wireless electronics

## Abstract

Humidity, along with temperature, is one of the most important environmental variables in people’s lives. The control of humidity is an important matter that is related to material properties and stability in various industries, as well as basic living. In order to detect humidity, changes in the physical, chemical, and electrical properties of materials related to humidity are used, and studies using various methods are conducted. In this study, a field-effect transistor (FET) device was fabricated on a soft polymer substrate with SnO_2_ nanowires (NWs), whose electrical properties change in response to water molecules. The SnO_2_ NWs, synthesized by chemical vapor deposition (CVD), were transferred onto a polymer substrate, using a sliding transfer method. The NW FET device, which was connected to an aluminum (Al)-based radio frequency (RF) receiving antenna, was wirelessly operated as a humidity sensor, based on the change in electrical properties of SnO_2_ NWs according to the relative humidity (RH). It was configured with a wireless antenna and light emitting diode (LED) indicator to implement a soft wirelessly powered humidity sensor that senses high RH and is expected to be used as a wearable electronic/sensor in the future.

## 1. Introduction

Humidity is generally measured as the relative humidity (RH), using hygrometers, including (i) the hair hygrometer that measures the changes in mechanical properties, mainly length, of the material by humidity [1]; (ii) the dry and wet bulb hygrometer that measures evaporation heat by evaporation [2,3]; (iii) the quartz oscillator dew point hygrometer that measures oscillation frequency change of piezoelectric quartz [4]; (iv) the capacitive humidity sensor that measures the change in capacitance of polymer or ceramic films [5,6,7,8,9]; and (v) the nano material-based humidity sensor that measures the resistance change of metal oxide nanomaterials [10,11,12,13,14,15].

Among these, the methods that measure the change of capacitance and resistance, which can be implemented in a thick- or thin-film form and utilize electrical characteristics, are the fields that have been studied most frequently. The former method measures the change in minute capacitance by water molecules adsorbed on the surface of the material. It is mainly composed of thin or thick film layers and has been studied in a variety of ways, from single layers to heterogeneous multilayers [7,16,17,18,19]. The latter method, which measures the resistance change of nanomaterials and calculates humidity, uses a simple structure that shows the electrical resistance change of a material depending on the water molecules and uses a more complex field-effect transistor (FET) structure that can enhance the changes in the resistance range due to an electrical field.

As the number of water molecules adhering to the surface increases, sensitivity is improved, so it is often implemented in the form of a one-dimensional (1D) nanowire (NW) or 2D thin film with a porous structure. SnO_2_ and ZnO have been widely used as metal oxide nanomaterials [10,11,13]. Among them, humidity sensing using SnO_2_ NWs is known to be able to measure the resistance change with high sensitivity by changing the depletion region caused by oxygen elements and external oxygen molecules on the surface of SnO_2_ NWs [10,20,21]. Recently, a great deal of research has been conducted to implement a wearable type of sensor, which has evolved from a conventional bulk-type humidity sensor to a flexible thin-film type, and has been used for personal respiration monitoring or skin attachable electronics that analyze the moisture content of the skin [22,23,24,25,26]. Table 1 summarizes the various humidity sensors that have a flexible form and a commercial one, as well. In this study, a soft, wirelessly powered humidity sensor that wirelessly drives a SnO_2_ NW FET, fabricated on a flexible soft polymer substrate, was fabricated, and the resistance varying greatly with RH was confirmed.

## 2. Materials and Methods

### 2.1. SnO_2_ NW Synthesis

**Figure A1:**
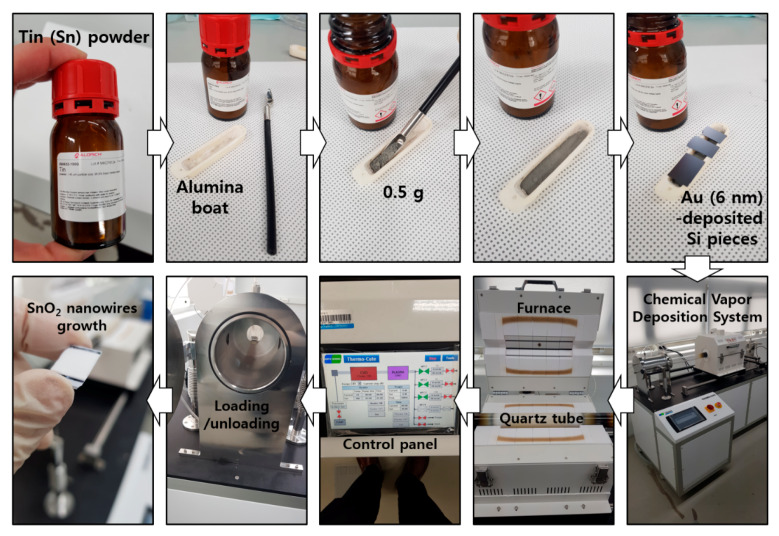
Synthesis procedure of SnO_2_ NWs, using CVD system. SnO_2_ NWs were grown on Au-deposited Si samples. Samples were loaded inside the Quartz tube with 0.5 g of tin powder in an alumina boat. After raising the temperature of the furnace up to 750 °C, SnO_2_ NWs were vertically grown for 30 min.

**Figure A2:**
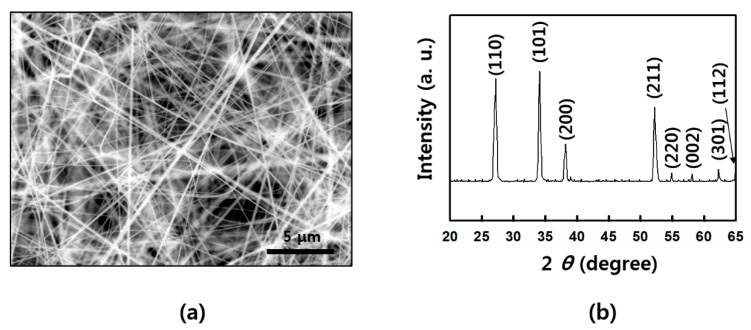
Structure of as-grown SnO_2_ NWs: (**a**) scanning electron microscope (SEM) image of SnO_2_ NWs; (**b**) X-ray diffraction (XRD) data of a rutile SnO_2_ NWs.

SnO_2_ NWs were prepared by chemical vapor deposition (CVD) via vapor–liquid–solid (VLS) process [27]. First, a sample prepared by depositing Au 6 nm was used as a catalyst on the cut silicon wafer by employing sputtering equipment. Approximately 0.5 g of tin (Sn) powder (Aldrich, St. Louis, MO, USA) was placed in an alumina boat, and the Au-deposited silicon wafer was placed on it. The alumina boat was inserted in the furnace chamber, and a mechanical pump was used to ensure a vacuum of up to 10^−3^ Torr. After the chamber temperature was raised to 750 °C over 30 min, the oxygen gas was added with 20 sccm, and the NWs were synthesized for 30 min. After completion, the sample was slowly cooled for about 2 h and then removed from the chamber. The synthesized SnO_2_ NWs had a diameter of about 80 to 110 nm and vertically grew from several hundred micrometers to several millimeters from a silicon substrate. The details of NW growth are presented in Figure A1. The structure of as-grown SnO_2_ NWs was analyzed by scanning electron microscope (SEM) (JEOL, Peabody, MA, USA) and X-ray diffraction (XRD) (Rigaku, Austin, TX, USA) in Figure A2, as well. SnO_2_ NWs shows a rutile structure in the XRD pattern (a lattice constant of a = 4.738 Å, b = 4.738 Å, and c = 3.188 Å) [28].

### 2.2. Substrate Preparation

The substrate was prepared by placing a polydimethylsiloxane (PDMS) thin film on a glass substrate. PDMS was made by mixing Sylgard 184 (Dow Corning, Midland, MI, USA) A with B in a ratio of 10:1, removing bubbles, and spin-coating at 2000 rpm on a glass substrate for 30 s. Subsequently, the PDMS substrate was thermally cured on a 110 °C hotplate for 30 min.

### 2.3. NW Transfer

**Figure A3:**
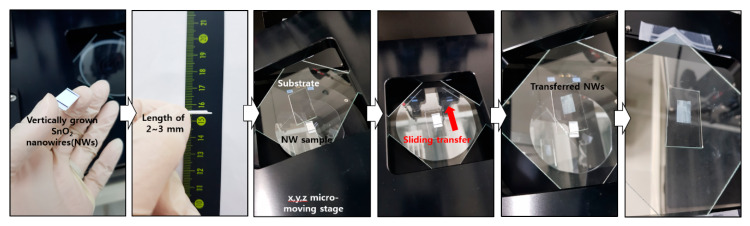
Sliding transfer method. The x, y, z moving stage was used to transfer NWs onto the desired substrate.

NW growth with CVD is not a cost-effective or energy-saving method when compared with other synthetic processes, such as a sol-gel method. A CVD normally needs high temperature circumstance, a few hundreds of Celsius degrees, for growth of nanomaterials with an expensive furnace for heating the chamber. Most functional devices mainly consisted of nanomaterials, especially 1D nanostructures, and required aligning and/or patterning techniques, as well as synthetic methods. Aligning the direction of NWs or patterning them to a desired size makes it possible to avoid the non-uniform performance distribution of the nanodevices as much as possible. The synthesized NWs use a sliding transfer method when being transferred onto a substrate [28,29]. This sliding transfer method can be used to align NWs which were grown by CVD, using simple physical methods. This is why SnO_2_ NWs were prepared in this study by using CVD. After the NW-grown silicon surface was attached to the substrate to be transferred, the NWs were transferred by horizontal sliding, with a distance of 3 mm, at a speed of 1 mm/min, while pressure (~1 kg/cm^2^) was maintained. The NWs were laid down on the substrate, in the sliding direction, while the degree of alignment and the length and density of the transferred NWs were determined by pressure, pushing distance, NW growth density, NW length, and substrate to be transferred. Alignment and length of the transferred NWs are important variables to ensure uniform electrical properties of devices, using NWs as a channel material. It is helpful to align the NWs with proper pressure during the sliding transfer process, but the large pressure shortens the length of the NWs and makes lots of NW networks between the electrodes. The longer the pushing distance, the shorter the length and less alignment, which can be solved by adding lubricant during sliding process. Moreover, the major variable to make sure good alignment and longer length of transferred NWs is the length of as-grown NWs. The image of millimeter-scaled NWs which were grown on the Si substrate can be founded in Figure A3. Density of transferred NWs is also variable to determine the electrical property of NW FET. However, it is quite related with the adhesive force and surface energy between the NWs and the transferred substrate. Additional surface modification is helpful to control the density of transferred NWs, but we did not perform it in this study. The sensor device we used, which has a channel length of 40 μm, experimentally confirmed the values of the pushing pressure of ~1 kg/cm^2^ and the pushing distance of 3 mm, through the fabrication of devices with variable parameters. More information about sliding transfer using a x, y, z moving stage is shown in Figure A3.

### 2.4. Device Fabrication Procedure

**Figure 1:**
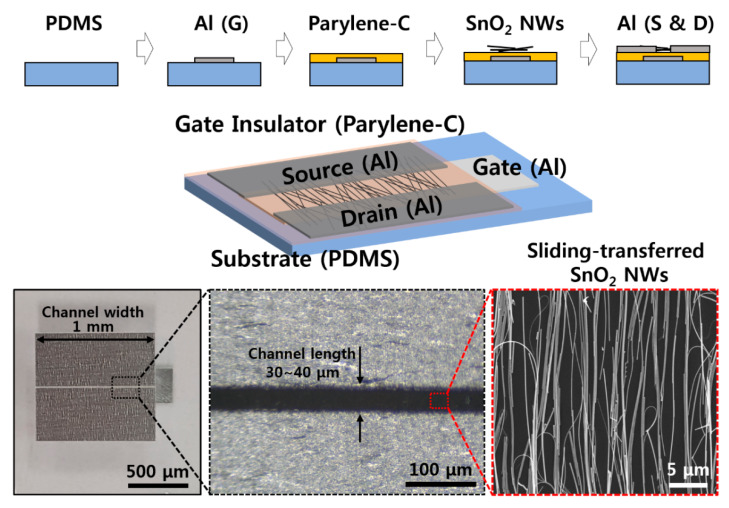
Schematic illustration of fabrication procedure and images of the SnO_2_ nanowires (NWs) field-effect transistor (FET) device.

Figure 1 shows the fabrication process for a SnO_2_ NW FET device. First, an aluminum (Al) gate pattern was fabricated on a cured PDMS substrate by CO_2_ laser marking equipment (Laser Marker, Hyosung Laser, Bucheon, Korea), and then a Parylene C thin film to be used as a gate insulator was formed, using CVD equipment (LAVIDA 110, Femtoscience, Hwasung, Korea). Di-chloro-di-p-xylylene (Daisan Kasei, Chiba, Japan) was used as the source, and the Parylene C thin film was formed by pyrolysis and deposition at ~700 °C for 20 min. An Al pattern, using laser equipment, and a SnO_2_ NW pattern aligned in a single direction, using a NW sliding transfer method, were fabricated on top of Parylene C. The density of the sliding-transferred SnO_2_ NWs used here was determined by SEM imaging at 1–2 NWs/μm based on the vertical direction of the alignment. The density and the length of transferred NWs determine the absolute value of the on/off current of the FET device and, here, one considers the related variables, the width and length of the channel, the power transfer properties of the transmitting and receiving antennas, and the RF output power.

**Figure A4:**
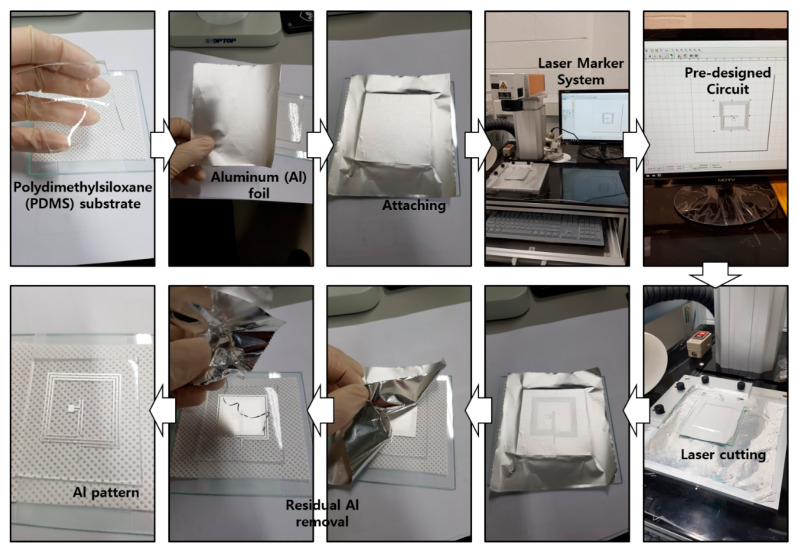
Fabrication procedure of the aluminum (Al) circuit pattern. The Al foil was etched with a predesigned pattern, using a laser marking system. Finally, residual Al was removed.

**Figure A5:**
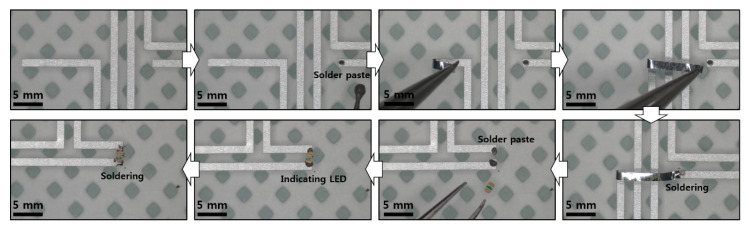
Microscopic images of chip integration and Al connection. A fine tweezer, sharp needle, and soldering gun with a sharp tip were used to connect the circuit.

Finally, the electrodes to be used as source and drain were prepared with an Al pattern in the same way. The soldering paste (SMD290SNL250T5, Chip Quik, Ancaster, ON, USA) was added to the contact area between the SnO_2_ NWs and the source and drain electrode due to metal particles that help the electrical connection between the NWs and the Al through heat treatment. The device was completed by O_2_ plasma treatment, to increase the sensitivity depending on the humidity [20,21]. SnO_2_ NWs showed little variation of electrical characteristics with the same growth temperature, oxygen gas amount, cooling method, and tin powder amount. In the case of the NW FET used as a sensor, at least 10 to 20 devices were manufactured with NWs grown under the same conditions, and the error bars in here represent a 95% confidence interval. More details of the fabrication procedure are presented in Figure A4 and Figure A5.

### 2.5. Wireless System

In order to manufacture a wirelessly driven humidity sensor, a near field communication (NFC)-based wireless power transmission system was utilized. Therefore, the humidity sensor device receives a signal from a 13.56 MHz radio frequency (RF) antenna, a SnO_2_ NW FET device whose resistance varies depending on humidity, and a circuit connected to a light emitting diode (LED) indicator to notify the operator of a change in resistance caused by humidity. A receiving antenna, an FET electrode, and an LED connection electrode pattern were manufactured by patterning Al on the same plane as the laser, and then soldering a red LED (SML-D12U1WT86, Rohm Semiconductor, Japan) using soldering paste to finish. The wireless power transmission system utilizes the Neurolux system manufactured by Neurolux (Chicago, IL, USA) and can control a space of 1 ft^3^ or more in two or three dimensions wirelessly with an output power of up to 10 W, depending on how the transmitting antenna is configured [30,31]. Here, a 5 cm × 5 cm receiving antenna is fabricated to drive the SnO_2_ NW FET, and the transmitting power is set to 4 W.

### 2.6. Humidity-Sensing Setup

**Figure A6:**
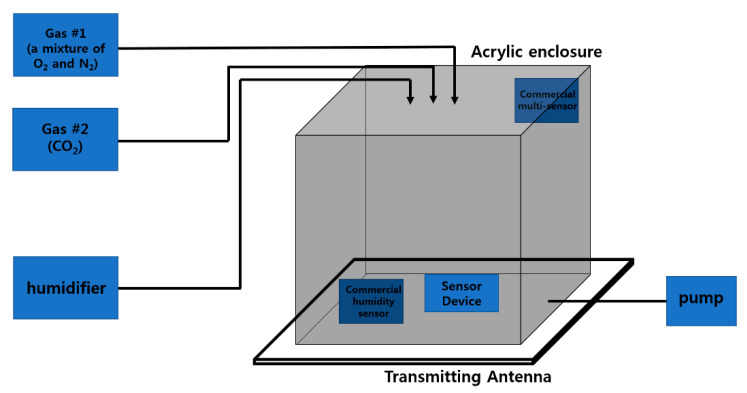
Schematic illustration of experimental setup for sensing of RH.

The humidity-sensing experiment was conducted as follows. After manufacturing the homemade acrylic enclosure, oxygen (O_2_) and nitrogen (N_2_) mixture gas lines, carbon dioxide (CO_2_) gas line, water molecule providing line, and pump line were connected. O_2_ and N_2_ gases were mixed in a 1:3 ratio. A RH was controlled through a humidifier and proceeded in the direction of increasing humidity. In order to check a RH and CO_2_, commercial sensors (G77597, Giltron, Taiwan and AR-837, Smart Sensor, GuangDong, China) with two different sensitivities were placed at the top and bottom of the enclosure, respectively, and variables influenced by other gases were excluded as much as possible through a mechanical rotary pump. The enclosure was placed above the transmitting antenna for the wireless operation. For the experiment, a pump and a mixture of O_2_ and N_2_ gases were used to remove other gases for 10 min, and an RH was lowered to 5% or less before the experiment started. Schematic illustration of setup can be founded in Figure A6.

## 3. Results and Discussion

### 3.1. Electrical Characterization of SnO_2_ FET

**Figure 2:**
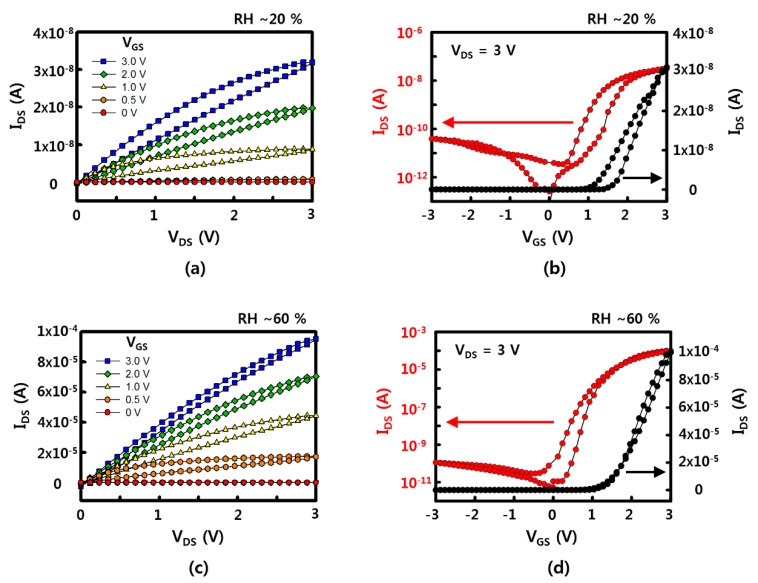
Electrical properties of a SnO_2_ NW FET device: (**a**,**c**) the drain-source current (I_DS_)–drain-source voltage (V_DS_) curve with variation of gate-source voltage (V_GS_) with a relative humidity (RH) of ~20% and ~60%, respectively; (**b**,**d**) the transfer curve of I_DS_-V_GS_ at V_DS_ = 3 V with a RH of ~20% and ~60%, respectively.

**Figure A7:**
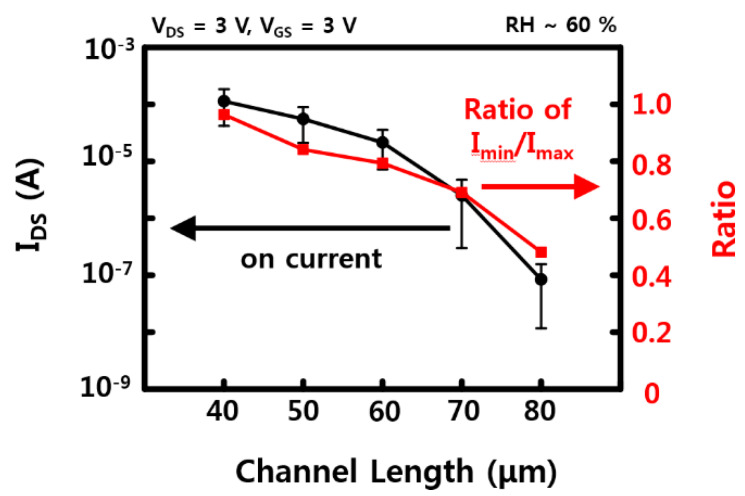
On current and the ratio of on currents between the devices which show the lowest on current and the device which shows the highest on current as a function of channel length, using 10 devices for every channel length. The error bars represent a 95% confidence interval.

Representative electrical characteristics of SnO_2_ NW FET devices fabricated by the sliding transfer method on soft and flexible PDMS substrates are shown in Figure 2. The FET devices show typical n-type semiconductor characteristics in which the drain-source current (I_DS_) increases when the drain-source (V_DS_) and gate-source (V_GS_) voltages are positive. In addition, the current value increases with strong gate-dependence as gate voltage increases from 0 to 3 V. The device used here has a NW channel width of 1 mm and a channel length of 30 to 40 μm, as shown in the images in the bottom of Figure 1. There are cases where it is connected through SnO_2_ NWs at once and a network of several NWs between source and drain. The network of NWs between the source and the drain electrodes could affect the electrical property and the variation between the sensors, as well. To minimize the effect of NW network, i.e., NW-NW contact, longer NWs are preferred to transfer. A shorter channel length can have less of an effect on the variation of the sensors, as well. The minimum channel length of FET, in here, was about 40 μm due to the diameter of laser. Additional on current and device variation data (with 10 different devices) with different channel lengths are shown in Figure A7. When the channel length increased, the on current decreased, while the ratio of on currents between the devices which show the lowest on current and the device which shows the highest on current as a function of channel length rapidly decreased. It showed that the uniformity of NW devices could be affected by the NW-NW contacts and shorter channel length will help to maintain uniformity of electrical properties. The on/off current ratios of the NW FET devices with an RH of ~60% and ~20% are ~10^6^ and ~10^3^ when V_GS_ = ±3 V based on V_DS_ = 3 V, and the on-currents are 10^−4^ and ~10^−8^ A, respectively. Based on this, the field effect mobility of the device with a RH of ~60% is similar to the previously reported SnO_2_ NW device that calculated the usage of a cylinder on a plane model at ~70 cm^2^/V·s [21]. This device is confirmed under the condition of 60% RH after O_2_ plasma treatment, which shows better gate dependence FET characteristics under the condition of higher RH after O_2_ plasma treatment compared with that of as-synthesized SnO_2_ NW FET [20,21].

### 3.2. Gate Dependence vs. Relative Humidity

**Figure 3:**
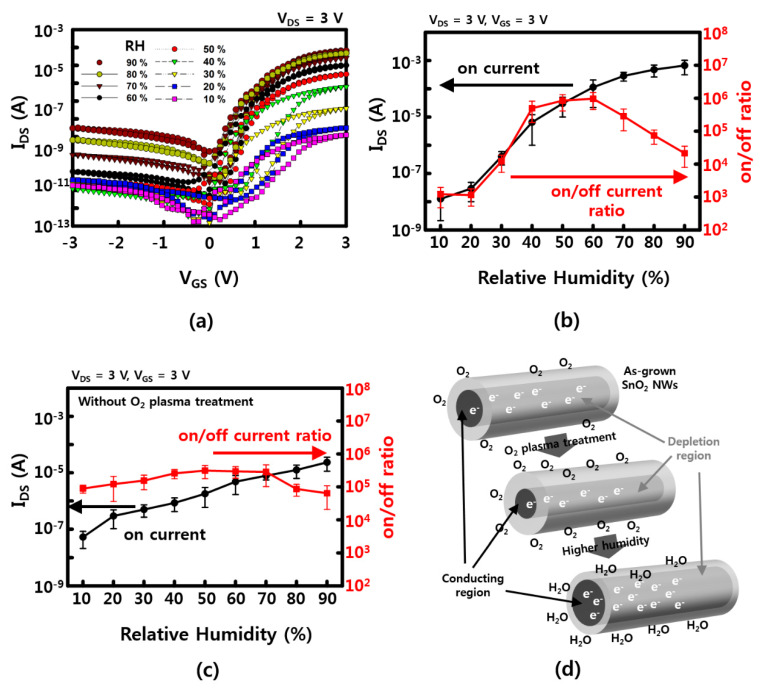
Humidity-related gate dependence (**a**) transfer curve of I_DS_–V_GS_ with 10%, 20%, 30%, 40%, 50%, 60%, 70%, 80%, and 90% humidity; (**b**) on current and on/off ratio as a function of RH after 3 min of O_2_ plasma treatment; (**c**) on current and on/off ratio as a function of RH without O_2_ plasma treatment; (**d**) humidity-sensing mechanism for the SnO_2_ NWs FET device. The error bars in (**b**,**c**) represent a 95% confidence interval.

V_GS_–I_DS_ characteristics are confirmed by increasing the RH to 10%, 20%, 40%, 50%, 60%, 70%, 80%, and 90% to compare the gate dependence effect. As shown in Figure 3a, the on current (at V_GS_ = 3 V) which is 10^−8^ A at 10% RH, is almost exponentially increased up to ~10^−4^ A at 90% RH. However, the off current is increased as a function of RH, as well. The on/off current ratio has a maximum value of ~10^6^ at 50% to 60% of RH. This can be explained by possible current leakages and/or less gate-effective depletion due to excessive water molecules that are absorbed on the NW surface. This leakage can be estimated with higher current values (I_DS_ ~ 10^−9^ A at V_GS_ = 0 V, V_DS_ = 3 V) at a RH of 90%. The values of the on current and the on/off ratio of SnO_2_ NW FETs, which vary with humidity, are presented in Figure 3b,c. In the case of SnO_2_ NWs, an n-type semiconductor has electrons as major carriers, and a 1D nanostructure has a large surface/volume ratio, which is affected by the adsorption–desorption of various gas molecules, including water molecules near the surface. When the O_2_ plasma is treated, the depletion region of the NW surface is increased by the physical collision of energetic oxygen atom/molecules on the surface and the O_2_ molecules adsorbed on the surface. Figure 3c shows the on current and the on/off ratio of O_2_ plasma-untreated NW FETs. The on current of untreated devices at a low RH between 10% and 20% is higher than that of O_2_ plasma-treated devices because the surface did not affect by collision of energetic O_2_ molecules yet. Although the on current was increased as a function of RH, the sensitivity (<10^2^) of untreated devices, which is varies with an RH of between 20% and 60%, showed to be much lower than that of O_2_ plasma-treated devices (~10^4^). Furthermore, the channel region, which is a passage through which the major carrier electron can pass, is reduced (see Figure 3d). At this time, the number of water molecules in the vicinity increases the probability that the water molecules are adsorbed on the surface of the NWs in place of the O_2_ molecules adsorbed on the surface of the NWs as the humidity increases, thereby reducing the depletion region enlarged by the O_2_ molecules. In addition, the channel is made large by the positive voltage (gate field), so that the electron can pass more quickly than the case without gate voltage [20,21]. Similarly, SnO_2_ NWs are known to be used as gas sensors that can detect a variety of gas molecules, including water molecules, such as carbon monoxide, carbon dioxide, ammonia, and nitrogen dioxide.

### 3.3. Wireless Sensor Application

**Figure 4:**
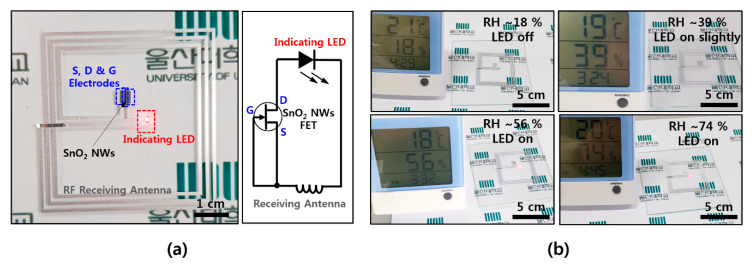
Wirelessly operated humidity sensor (**a**) NFC-based wireless humidity sensor device and its circuit diagram; (**b**) humidity-sensing operation with red LED indicator as a function of RH ~18%, ~39%, ~56%, and ~74%.

In order to utilize the wearable electronics as a humidity sensor, a wirelessly driven SnO_2_ humidity sensor was fabricated on a thin film of a flexible soft material. An antenna (width: 200 μm, spacing: 200 μm, overall size: 5 cm × 5 cm) that can receive an RF signal with a frequency of 13.56 MHz, which is the communication standard of NFC, was configured and connected to the source and gate electrodes of the FET device, in order to be operated wirelessly without a battery. When the RF signal is received with an antenna, the gate increases the conducting region and the current flows sufficiently to operate the LED indicator. Figure 4a shows the overall shape of the device and shows a circuit consisting of a receiving antenna, an FET device, and an LED indicator. A transmitting power of 4 W and a distance of 5 cm from the transmitting antenna are required for the 5 cm × 5 cm receiving antenna, while an antenna with a width of 200 μm, spacing of 200 μm with three turns, NW FET channel width of 600 μm, channel length of ~40 μm, and density of 1~2 NWs/μm is configured. As shown in Figure 4b, the LED, which is not driven at an RH of ~18%, is driven from a RH of ~39%, and shows higher light intensity at a RH of ~56% and ~74%. It is confirmed that the operation range can be controlled by properties of the transmitting/receiving antenna and the dimension of the FET channel. In the case of humidity, it is difficult to change environmental conditions quickly, and the reaction rate is also known to be slower here than in other gas molecules. However, it is expected to be used as a humidity alarm for providing a warning of high humidity in wearable electronics, based on the results of this study. It is also expected to be easily applied to commercial card readers or smartphones with NFC by utilizing existing NFC-based wireless communication without an additional battery on the device itself.

**Figure 5:**
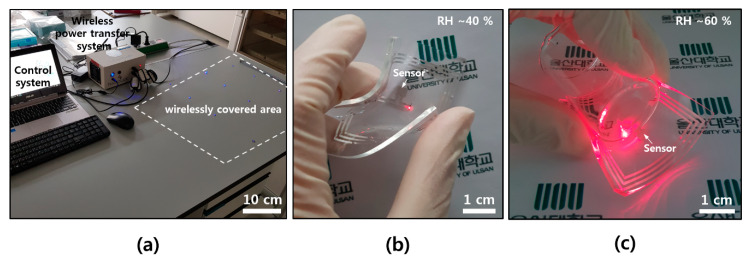
Photographs of wireless system and devices. (**a**) Wireless power transfer and control system with covering area; (**b**) wireless sensing operation at a RH of ~40% in a bent state; and (**c**) wireless sensing operation at a RH of ~60% after wrapping on the small beaker (diameter of ~3 cm).

For the wearable applications, a soft and flexible form of electronics is needed, as well as a wireless capacity. Unlike a conventional electronic flatform, our skin or clothes have lower modulus, and there might be a modulus mismatch between the animal body (~kPa) and the silicon electronics (~GPa) [30,31]. Figure 5a shows a wireless power transfer system, control equipment, and a wirelessly covered area by transmitting antenna that is setup underneath. A commercial near field wireless power transfer system, Neurolux in here, offered diverse expandability on the design of transmitting antenna for applying it to all kinds of experimental apparatuses [31]. The sensor devices with a receiving antenna pattern could be easily operated by a simple single roof antenna underneath the desk without additional impedance matching circuit on the devices. Humidity sensor devices, which consist of soft and flexible elastomers (PDMS) and ultra-thin Al, were wirelessly operated on top of a transmitting antenna, even in bent (Figure 5b) and wrapped (Figure 5c) states.

### 3.4. Device Stability

**Figure 6:**
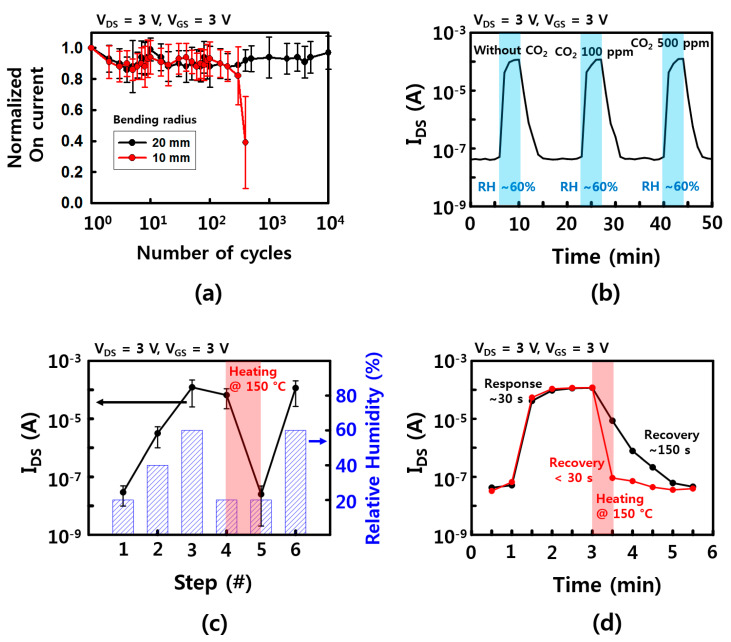
Device stability characteristics. (**a**) Mechanical stability of the sensor device. Normalized on current as a function of bending cycles with bending radius of 10, 20 mm; (**b**) Response and recovery time with/without CO_2_ gas (100 and 500 ppm); (**c**) the on current of the sensor devices depends on the condition of RH and the heating step for removal of water molecules; (**d**) response and recovery time with/without heating step. The error bars in (**a**,**b**) represent a 95% confidence interval.

Mechanical stability is one of the important properties for applying wearable and wireless platforms. The FET performance of fabricated humidity sensors was tested with bending deformation. In the fatigue test of Figure 6a, the devices can survive up to ~10^4^ and ~ 400 cycles with a bending radius of 20 and 10 mm, respectively. These devices are mechanically robust, but the deformable properties were not as good as other results [21,30,31]. This can be explained by the disconnection on the network of the NW channel between the source and drain electrodes during bending deformation. Shorter channel length or longer NWs, which are transferred between source and drain electrodes directly, will enhance the mechanical stability. Additionally, metal oxide NWs, including SnO_2_, normally have robust chemical and thermal stabilities due to their high processing temperature, high melting point, and stable atomic structure [10,21]. There was no noticeable degradation of device performance during the entire time (a few months) of these experiments. Moreover, SnO_2_ NW is sensitive to many different gases because the electrical properties of the surface change as the oxygen molecules on the surface are replaced with other gases. Therefore, a cross-check was performed to see if other gases were influencing, and in here, CO_2_ was used to check the change in on current according to RH. As shown in Figure 6b, when the humidity was changed from 20% humidity to 60% humidity at room temperature, there was no noticeable change when the on current without CO_2_ and with 100 and 500 ppm of CO_2_, respectively. One of the reasons is that the concentration of CO_2_ is relatively low, so it has little effect on the increase in on current than the effect of RH change. Moreover, the temperature at which CO_2_ is adsorbed at the maximum is much higher than room temperature [32]. Considering that many other gases have a high temperature for the maximum adsorption, the humidity sensor which is operated at room temperature may be less affected on the other gases. However, humidity sensors, like other gas sensors, need a recovery step to eliminate pre-absorbed molecules. The on current of our devices showed RH dependence characteristics when RH was increased up to 60%, while there was no noticeable change of the on current for a short time after RH was lowered down to ~20%. This was because the absorbed water molecules when RH was increased were still attached on the surface of the NWs at the low RH. To avoid this sensing issue, an additional heating process was used to remove the pre-absorbed water molecules. After 150 °C heat treatment on a hot plate for a minute, the devices fully recovered their electrical property and reusability, as shown in Figure 6c. As shown in Figure 6d, the recovery times with RH between 60% and 20% were measured as ~150 and <30 s, with/without additional heating step, respectively. Although there are no results on the wireless heating device yet, the micro-heater device with a thin flatform, which can be operated with a wireless system, will be an important part of wireless and wearable sensor applications.

## 4. Conclusions

A SnO_2_ NW FET device, whose electrical properties change with humidity, was fabricated on a flexible polymer substrate, as a humidity sensor. Sliding transferred NWs showed strong gate dependence characteristics as a function of RH. The sensor devices were prepared with well-developed procedures that do not need any cleanroom facilities, while the devices showed robust mechanical stability and reusability. By using this, it became possible to implement a humidity sensor that can be wirelessly driven and applied to wearable electronics. Subsequent research is expected to be used in IoT-based wearable sensors in order to improve the adsorption–desorption reaction speed upon adding a micro heater and smart sensor control through the introduction of NFC chips.

## Figures and Tables

**Table 1:** Flexible and commercial humidity sensors.

Properties	This Work	Pang et al., 2018 [22]	Zhang et al., 2019 [23]	Yang et al., 2019 [24]	Jeong et al., 2019 [25]	Zhou et al., 2020 [26]	Commercial Sensor SHTC3 ^8^
Sensor type	Field-effect transistor	Resistive	Resistive	Resistive	Resistive	Resistive	Capacitive
Sensing material	SnO_2_ nanowire (NW)	Porous graphene	Ag coated Fe_3_O_4_ NW	MoO_3_ nanosheet	Au coated PVA ^1^	PEDOT: PSS	Unknown
Process	CVD ^2^	CVD	Solution growth	Solution growth	Electrospinning	Nano- confinement	Unknown
Purpose	Humidity alarm	Respiration monitoring	Respiration monitoring	Noncontact touch sensor	Real-time sensing	Respiration monitoring	Sensor chip
Sensitivity ^3^	~10^4^	<10^2^	~10^3^	~10^3^	<10^2^	<10^2^	~0.1% RH
Recovery	Slow	~72 s	~75 s	<1 s	~110 s	~41 s	~8 s
Substrate	PDMS ^4^	PET ^5^	PP ^6^	PET	PVA fiber	PDMS	Epoxy resin
Wireless availability	NFC ^7^-based wireless operation	-	-	Bluetooth available	-	Bluetooth available	Battery included

^1^ Poly (vinyl alcohol). ^2^ Chemical Vapor Deposition. ^3^ Resistance (or current) change depending on relative humidity of between 20% and 60%. ^4^ Polydimethylsiloxane. ^5^ Polyethylene terephthalate. ^6^ Polypropylene. ^7^ Near field communication. ^8^ Digital humidity sensor (SEK—SHTC3, Sensirion, Chicago, IL, USA).

## Appendix A

Figure A1 shows the detailed procedure for preparing the SnO_2_ NWs using CVD. Figure A2 shows the structure of as-grown SnO_2_ NWs. The sliding transfer method that was used here is shown in Figure A3. Figure A4 and Figure A5 represent the detailed fabrication and soldering procedures, respectively. The experimental setup for humidity sensing is shown in Figure A6. Figure A7 shows on currents as a function of channel length.
